# Chiropractic students’ experiences on the use of virtual radiography simulation: a pilot observational study

**DOI:** 10.1186/s12909-021-02827-0

**Published:** 2021-07-28

**Authors:** Madeleine Shanahan, Tom Molyneux, Dein Vindigni

**Affiliations:** 1 School of Health Sciences, Faculty of Health, University of Canberra, Canberra, Australia; 2 School of Health and Biomedical Sciences, RMIT University, Melbourne, Australia

**Keywords:** Chiropractors, Radiography, Simulation

## Abstract

**Background:**

Virtual radiography provides students with an opportunity to practise their clinical skills in patient positioning and evaluating radiographic images. The purpose of this pilot study was to introduce Projection VR™, a software radiography simulation program, into a student chiropractic program and evaluate its potential application as a teaching and learning tool.

**Methods:**

Undergraduate chiropractic students, enrolled in a radiographic course (unit within the chiropractic program), were invited to attend a scheduled laboratory where they were introduced to, and undertook purposefully designed activities using the radiography simulation. At the end of this activity, students were asked to complete an online survey (see Virtual Radiography Survey) to describe their experiences of the educational value of the software program. Descriptive statistics were used to evaluate outcomes. Content analysis was performed for free-text comments provided by respondents with key themes provided by the predetermined quantitative categories of the questionnaire.

**Results:**

Responses were received from 44 out of the 47 students who attended the scheduled laboratory (response rate 92%). Overall students were positive about this simulation identifying that it was easy to use (95%) and that they could control the equipment as needed (95%). The main reported benefits included students being enabled to repeat tasks until they were satisfied with the results (98%) and being able to quickly assess images and determine if changes needed to be made (98%). Participants reported improvement in their understanding of the effect of exposure factors on patient radiation dose (93%) as well as their technical image evaluation (84%) and problem-solving skills (80%).

**Conclusions:**

The results of this study suggest that virtual radiography is a valuable complementary resource in providing chiropractic students with radiographic knowledge and skills.

**Supplementary Information:**

The online version contains supplementary material available at 10.1186/s12909-021-02827-0.

## Background

The World Federation of Chiropractic defines chiropractic as a health profession concerned with the diagnosis, treatment and prevention of mechanical disorders of the musculoskeletal system and the effects of these disorders on the function of the nervous system andAQ3 general health [1]. Radiography has been integrated into chiropractic teaching programs since 1910 when B.J. Palmer purchased an x-ray unit for the Palmer School of Chiropractic in Davenport, Iowa [2]. It has been used as a diagnostic tool in the biomechanical evaluation of the spine and pelvis and to help identify contraindications to manual therapy.

It continues to be taught as a core course in all chiropractic programs in Australia and most chiropractic programs throughout the world [3–5].

Maintaining the quality of imaging whilst minimising the radiation dose to patients is a priority which is highlighted by the Australian Radiation Protection and Nuclear Safety Agency [6] and one which is emphasised in clinical radiographic training and radiology courses (MEDS2144 Introduction to Diagnostic Imaging, MEDS2143 Advanced Diagnostic Imaging and REHA2203 Chiropractic 6 Theory) at RMIT University [7].

The opportunity to participate in simulated radiography complements rigorous training in the theoretical and clinical aspects of plain film diagnostic imaging. At the time of this pilot study, radiography was a subject in year four of the five-year chiropractic program. This was undertaken prior to the students performing supervised radiography in the teaching clinics in which students treat members of the public under the supervision of qualified chiropractors. Supervised radiography may be performed by the students as part of that experience. Students graduating from the chiropractic course are eligible to apply for a limited x-ray (Use) licence and so it is important that students have a sound understanding of the radiographic principles and practice that are related to the scope of practice of their limited x-ray licence.

In recent years, computer-based simulation radiography has been introduced in undergraduate radiography programs with promising results [8, 9]. However, its usefulness in achieving clinical learning outcomes in pre-clinical undergraduate chiropractic programs has not been previously piloted and evaluated.

This pilot study aimed to introduce a software radiography simulation tool called Projection VR™ into a chiropractic, pre-clinical undergraduate setting and gather student feedback about its application as a clinical learning tool and strategy. According to the literature, no other chiropractic institution or training program has implemented simulated radiography in their curriculum. Since completing this pilot study; however, literature has been published on the use of other virtual radiography software [10, 11]. The Projection VR TM software has been well described by Shanahan [9].

## Methods

The Human Research Ethics Committee of the Royal Melbourne Institute of Technology (RMIT) University (BSEHAPP 06–15) approved the project, including its design and recruitment methods.

### Research design

This descriptive study used an observational pilot to evaluate the impact of the software. In this pilot study, students had their first introduction to the Project VR TM software. As this was an initial implementation, we sought to obtain student quantitative and qualitative feedback as part of the evaluation process.

### Participants

Chiropractic students in the second semester of year four of the program in 2016 were recruited as part of their traditional practical sessions in radiography. Informed consent was obtained in the first test item of an online survey (see Virtual Radiography Survey), with respondents able to exit at this point if they so preferred. Of a total of 113 students enrolled in the subject, only the first 20 in each of five groups were given the opportunity to participate. We used a convenience self-selection method to recruit the participants, forty four students voluntarily responded to the survey questions. Students involved in this pilot study had already spent 11 weeks of a 12-week semester with the radiography tutor.

### Procedure

Participating chiropractic students were scheduled to attend one laboratory session which used the computer-based virtual radiography simulation software Projection VR™. Projection VR™ simulation in this university setting could be adequately delivered via Windows 8 or 7 (64 or 32 bit) with a graphics processor of at least DirectX and Shader Model 3.0 or 4.0 hardware support and 512 megabytes or more of dedicated video RAM. The standard computer laboratory equipment at the university met or exceeded these requirements.

A worksheet on simulated radiography of the lumbar spine was developed for chiropractic students using Projection VR™. The lumbar spine was chosen as it represents one of the most common areas that chiropractors request radiography for. In a research paper by Metaxas (2018), the seven most common areas to be x-rayed included the lumbar spine and pelvis. No training was conducted prior to using the simulated radiography system as students learned to use the technology while they undertook the activity under the guidance of an experienced lecturer in radiography [12]. The detailed worksheet allowed the students to use the technology as they undertook the laboratory activity. Each student used the simulation individually.

**Fig. 1:**
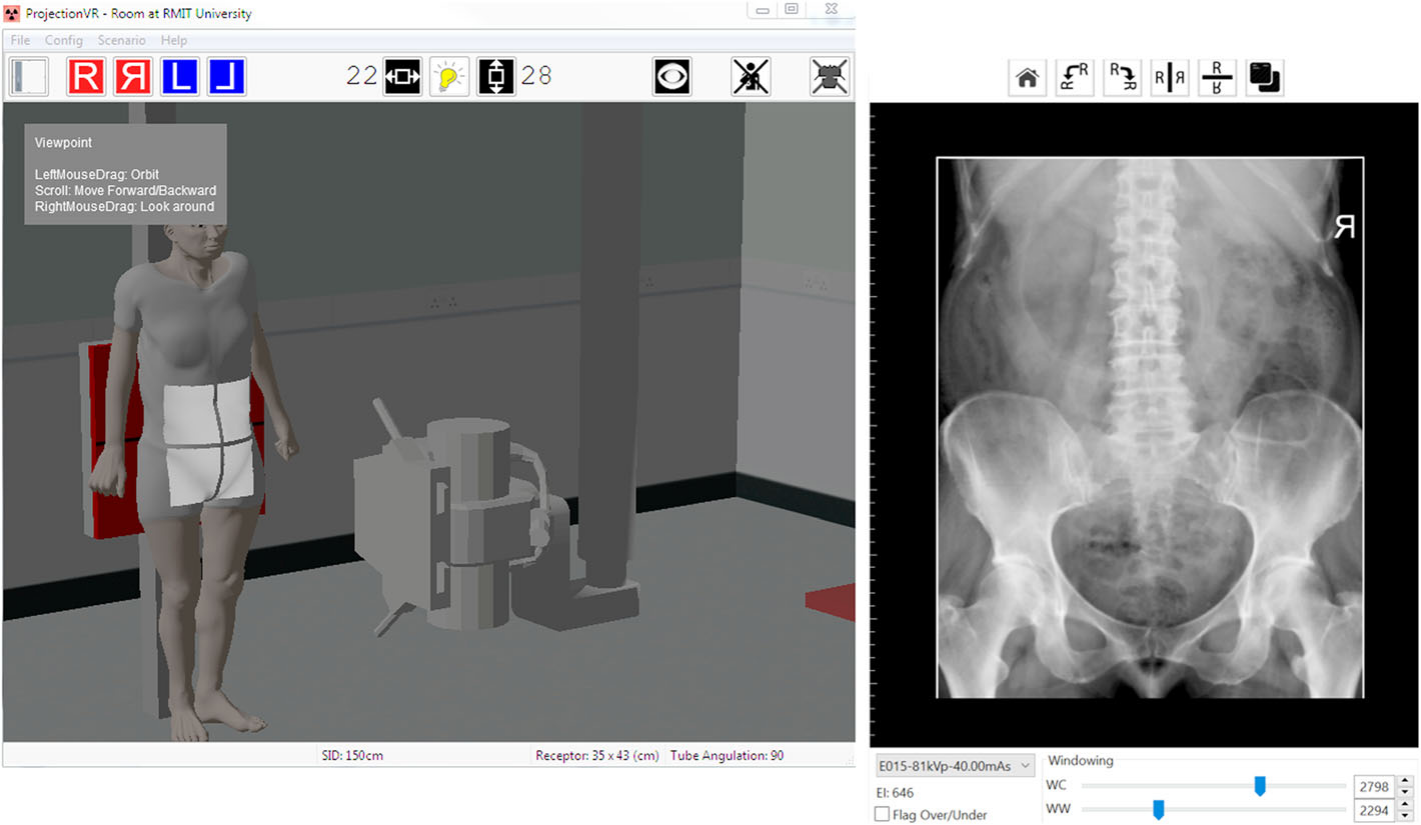
Technical set up for Anterior to Posterior lumbopelvic (left) and resultant image generated (right) using Projection VR™

**Fig. 2:**
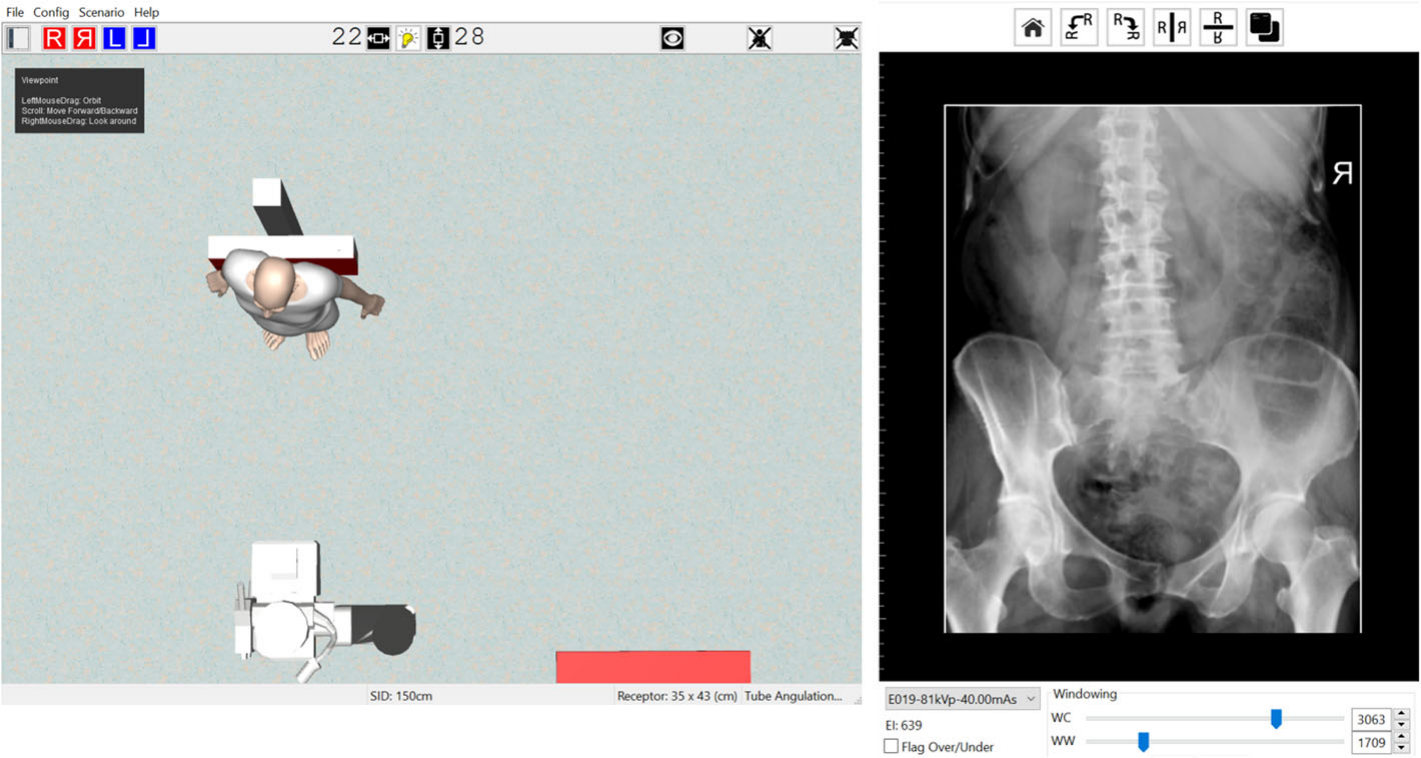
Technical set up for rotated (Right anterior oblique position) Anterior to Posterior lumbopelvic (left) and resultant image generated (right) using Projection VR™

**Fig. 3:**
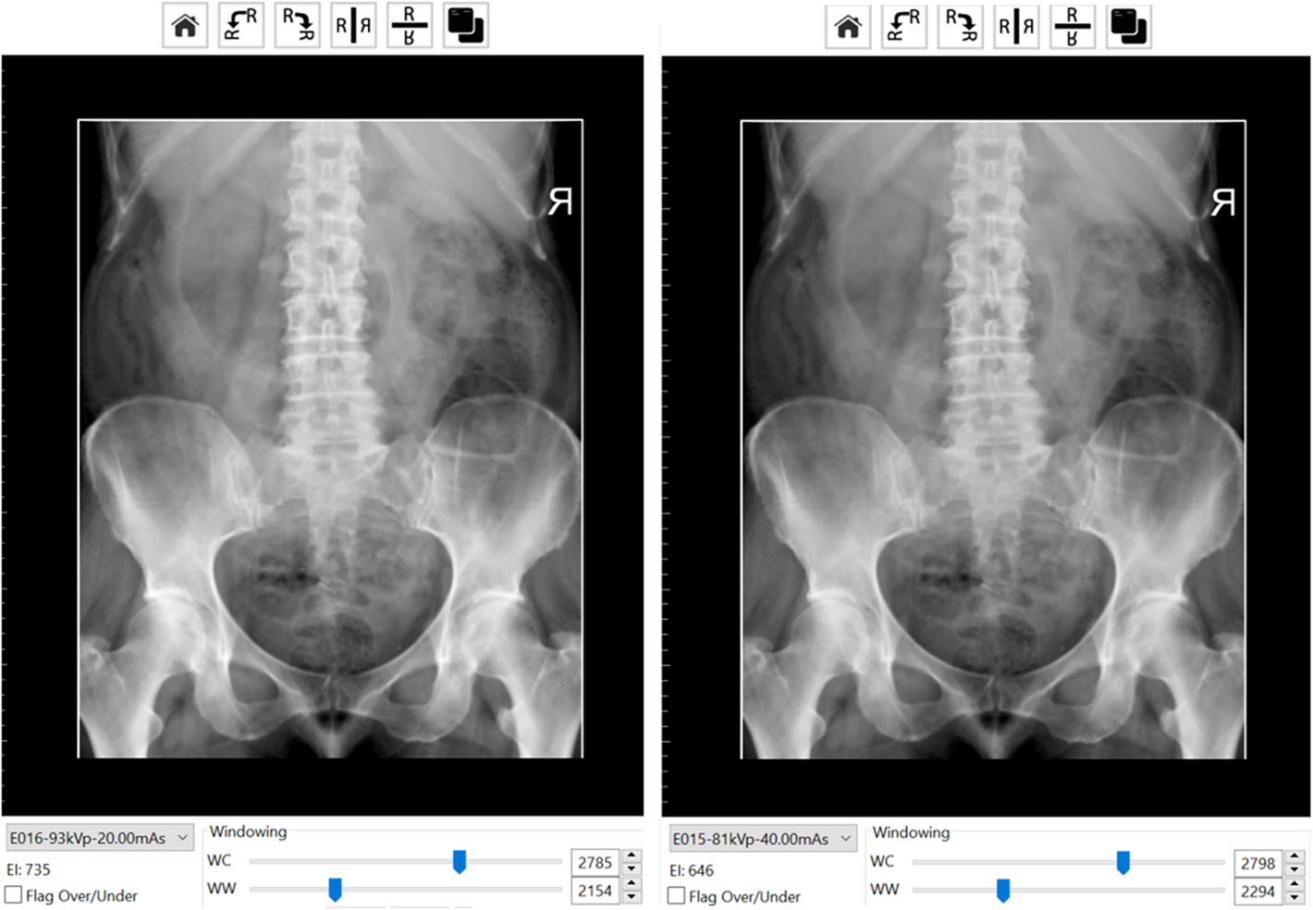
Images generated using Projection VR™ with 15% rule applied. Image on right 81 kVp, 40 mAs and image on left 93 kVp, 20 mAs

**Fig. 4:**
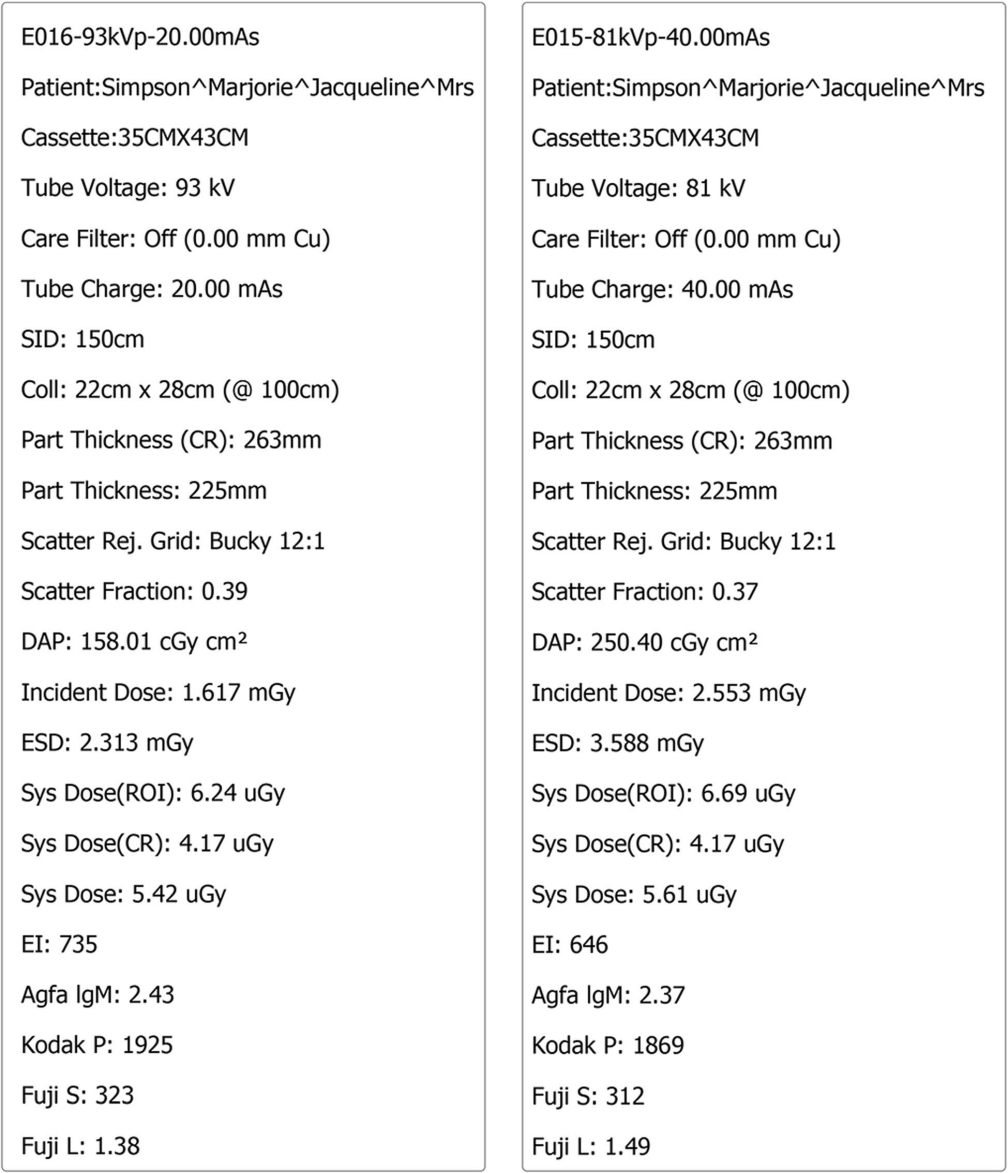
Technical data display available when using Projection VR™ allowing for comparison of Entrance Surface Dose (ESD) with 15% rule applied. Image on right 81 kVp, 40 mAs and image on left 93 kVp, 20 mAs

**Fig. 5:**
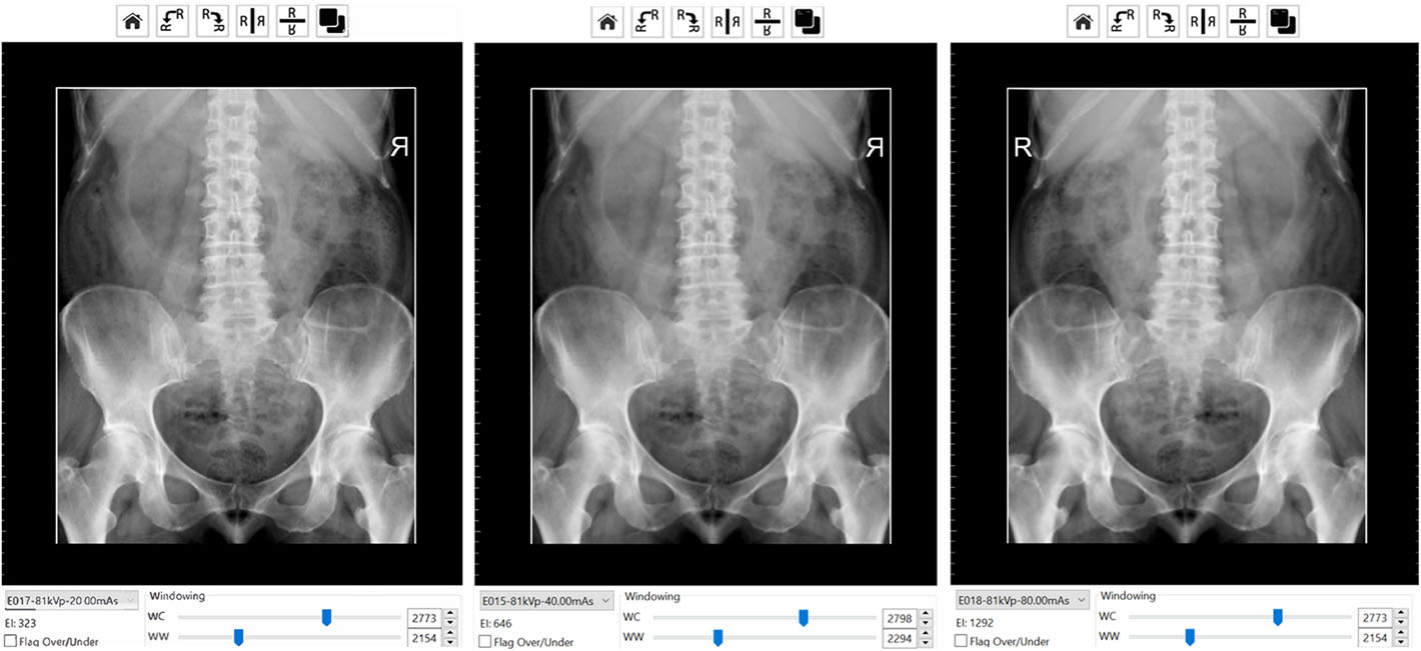
Images generated using Projection VR™ at 81 kVp and varying mAs 20 mAs (left), 40 mAs (centre) and 80 mAs (right)

There were three key areas of focus for this activity. Firstly, for each student to simulate patient positioning and technical set up in preparation for taking the Anterior to Posterior lumbopelvic image and to generate an AP lumbopelvic (APLP) image (Fig. 1). Secondly, having produced an unrotated APLP, students were then asked to rotate their patient so that the patient’s right posterior side was closer to the image receptor (Fig. 2). Before generating the image, students were asked what distinguishing anatomical features they expected to see on the image (Fig. 2). This strategy was used to support active and engage critical thinking as students consciously paused and reflected before they undertook their next action [13]. Thirdly, the effect of exposure factor selection on the digital image as well as patient dose was investigated. This was tested using two methods, namely application of the 15% rule and the effect of decreasing and increasing milliamp seconds (mAs) on digital images. Increasing kilovolt peak (kVp) with a concomitant decrease in mAs is expressed as the 15% kVp rule. The 15% rule states that a 15% increase in kVp is the equivalent of doubling the exposure received at the image receptor [14, 15]. To maintain exposure at the image receptor, the mAs is halved. The increase in kVp, when applying the 15% rule, is variable and dependent on the original kVp. For example, at 60 kVp, the calculated change in kVp would be 9 kVp whereas at 80 kVp, the required change would be 12 kVp. Studies examining application of the 15% kVp rule have demonstrated a considerable reduction in patient dose (22–60%) without adversely affecting image quality [16, 17]. This is an important finding as increasing kVp reduces subject contrast and could therefore potentially negatively impact image quality [14, 15]. Before generating the image acquired using the 15% rule, students were asked what change if any they may expect to see on the image (Fig. 3) and what impact, if any, applying the 15% rule would have on patient dose. Technical data available in Projection VR™ relating to radiation dose for the two images generated is provided in Fig. 4. Entrance surface dose (ESD) measurements were compared for the two exposure techniques. The final aspect of selection of exposure factors on digital images was undertaken by asking students what difference they would expect to see on the radiographic image and on patient dose if they were to half or double the mAs. Generated images are provided in Fig. 5.

Throughout the session, students were asked to predict the outcome of each change in patient position or exposure factors before they generated and saw images or technical data. This method was used to encourage students to think critically in applying their decision-making skills in a clinical setting and has been found to reinforce learning by other authors [13, 18, 19] .

### Data analysis

The survey data were entered into SPSS 21.0® and descriptive statistics were used for analysis. Analysis of survey data was descriptive only as this research involved an exploratory snap-shot process - that is, the survey was only administered once [20] .

## Results

Responses were received from 44 out of 47 participants in the practical sessions. The sex and age characteristics of the responding students are presented in Table 1.

**Table 1:** Demographic characteristics of participants (*n* = 44)

Characteristic		Number (%)
Sex	Female	30 (68)
	Male	14 (32)
Age (years)	18–21	22 (50)
	22–25	11 (25)
	26–29	4 (9)
	30 +	7 (16)

The majority of students were female (68%) (M = 14, F = 30). All age groups were represented in this sample and included both high school leavers and mature aged students.

### Technology

Given that Projection VR™ involves computer-based simulation, participants were asked about their confidence in using this technology as part of their learning strategy. Eighty-two% (*n* = 36) of responding participants described themselves as *confident* or *moderately confident* in the use of computer technology. Only 18% (eight students) reported that they had previous experience using computer-based simulation. Students’ experiences of ease of use of Projection VR™ are summarised in Table 2.

**Table 2:** Students’ experiences of ease of use of Projection VR™ (*n* = 44)

Question	Strongly agree	Agree	Neither agree nor disagree	Dis-agree	Strongly disagree	Total Number
I could control the equipment as I needed when using Projection VR™	15	27	2	0	0	44
I liked using Projection VR™	17	22	4	1	0	44
Projection VR™ is easy to use	8	34	2	0	0	44
Technical problems made using Projection VR™ difficult	4	6	11	14	9	44
The Projection VR™ laboratory activity sheet was easy to follow	7	29	7	1	0	44
***Using Projection VR™***						
Encouraged me to think more about radiographic procedures	17	22	3	2	0	44
Had a positive effect on my ability to set up a radiographic examination	15	17	8	3	1	44
Allowed me to quickly see images and understand if changes needed to be made	25	18	1	0	0	44
Had a positive effect on my ability to evaluate radiographic images	17	20	7	0	0	44
Helped me learn as I was able to repeat activities until I was satisfied with the results	21	22	1	0	0	44
Encouraged me to solve problems	12	23	8	1	0	44

### Qualitative feedback of students’ experiences of using projection VR™

Content analysis was performed for free-text comments provided by respondents with key themes provided by the predetermined quantitative categories of the questionnaire. Four themes emerged: ease of use of the simulation, learning using the simulation, limitations of the simulation, and recommendations for future implementation.

#### Ease of use of the simulation

Students were positive regarding ease of use of the simulation software and their ability to control the equipment during the learning activity. Some students commented that the simulation could be improved by making it easier to use, with one student specifying “some of the keys made it hard to set up accurately”.

#### Learning using the simulation

Students generally considered Projection VR™ as a worthwhile educational tool that quickly generated radiographic images and enabled them to refine the process until they were confident in its application. Student comments described benefits of using the simulation including “Easy and quick visualisation of an x-ray image”, “It was a good simulation to be able to see what we’re actually imaging” and “you can visualise anything you want to, at whatever angle you want to. Very helpful especially for visual learners like myself”.

Students also reported that using the program encouraged them to think more about radiographic technique and it facilitated their problem-solving skills. Student comments included “made me think about what I was doing”, “helped me to think wisely on how the image should be produced” and “enabled me to produce images and see where I needed to correct myself to get a better image”.

In addition, students identified that the simulation activity enhanced their understanding of technical factor selection and radiation dose. For example, students stated that using the simulation “helped me to understand the exposure”, “helped me understand the technical side, not just positioning” and it “gave us extra information on patient dose and levels of radiation”.

Students also described the use of this simulation program as enhancing their learning opportunities. Their comments included “it combines the theory and practical context taught during Chiropractic Theory 6 whilst introducing a new way of expanding our skills and knowledge through technology” and “regular practice sessions with the computers would be great to assist the physical learning”. Students also recognised that this simulation provided a safe learning environment as it did not require the use of radiation “being able to redo and correct any mistakes without worry”.

#### Limitations of the simulation

Students did report limitations of the simulation including that, as movement of x-ray tube was controlled by computer keys, “some buttons are difficult to find” and “some of the keys made it hard to set up accurately”. It was also noted that as you are unable to palpate the virtual patient “more bony landmarks on patient” were needed.

#### Recommendations for future implementation

Students in this study identified that remote access to this simulation would be a beneficial change, for example, ‘simulation of practicals into computers makes practice easier and more accessible’, ‘make it readily available to practise at home’ and ‘use of it at home via RMIT website, lists of views required for exam so we can practice’.

## Discussion

The aim of this study was to explore chiropractic students’ experiences of the Projection VR™ to assist in developing their radiographic skills and confidence in a laboratory setting. This study suggested that the simulation did improve students’ learning experience.

The Projection VR™ was previously incorporated into the Medical Imaging program in the School of Health and Biomedical Sciences (SHBS) at RMIT University with most technical complications being resolved by the time the simulation program was trialled with chiropractic students. In general, the program supported students’ skill development and enhanced confidence levels.

Another potential application of this program is remote access by students. The advantages of such access include the flexible delivery of learning and teaching, overcoming geographical barriers in terms of travel as well as students being able to acquire skills and knowledge at their own pace [21].

Given that there is variety in the reported levels of confidence, computer skills and abilities among students, the option of their being able to progress through simulation activities at their own pace is likely to facilitate the learning experience.

### Confidence and skill development

In general, the introduction of Projection VR™ increased students’ confidence in patient positioning procedures and their ability to evaluate radiographic images. It has been reported that enhancing students’ clinical radiographic skills as they make the transition from their pre-clinical undergraduate education to clinical practice may help to alleviate the stress associated with this transition [22].

Having acquired the skills to confidently set up radiographic procedures and evaluate images, students have reported being able to better focus their energies on refining their communication and patient-interaction skills [23].

Students also described that participating in the Projection VR™ simulation positively influenced their ability to problem solve. These findings are consistent with other published reports that highlight the value of students critically reflecting on their perceived strengths and weaknesses as a step to solving future clinical challenges and contributing to a range of other important clinical and professional standards [24, 25].

Ninety-three percent of students identified that the simulation activity enhanced their understanding of the effect of changing radiographic exposure factors on patient dose. Chiropractors who perform radiography have a responsibility to select exposure parameters which minimise patient dose when producing clinically diagnostic images. Key x-ray parameters that a chiropractor controls and can manipulate for radiographic examinations include tube voltage (kVp), tube current and time (mAs) and source to image distance (SID). As SID is traditionally fixed at 150 cm for chiropractic planar imaging [26], student chiropractors should develop a good understanding of 15% rule as a radiation dose reduction strategy. Projection VR™ simulation does provide similar percent dose reduction measurements to direct dosimetry measurements when assessing application of the 15% rule [27]. Projection VR™ is a useful educational tool to support student learning focussed on exposure parameters and dose reduction technique in planar radiography. Potential applications of the simulation program within the chiropractic curriculum may include:

- assist in providing a blended learning approach to teaching radiographic positioning that includes the theoretical basis of radiography in chiropractic practice, face-to-face practicals and virtual radiography to complement and reinforce these more traditional approaches to teaching and learning;
- help students to better understand how positioning influences radiation exposure (including factors such as skin dose and absorbed dose);
- provide a cost-effective and efficient mechanism to ‘practice’ positioning;
- assist in demonstrating how positioning influences radiographic anatomy;
- currently, none of the four chiropractic programs in Australia incorporate virtual radiography in their curricula and the preliminary findings of this study demonstrate the potential to incorporate virtual chiropractic radiography into their curricula as part of an effective blended learning approach to learning and teaching.

More broadly, Projection VR TM may be a valuable adjunct to health professionals who with suitable radiographic training may operate diagnostic x-ray equipment, as an example, due to large geographic distances, radiographic services in remote areas in Australia are often provided by appropriately trained remote x-ray operators who include nurses, general practitioners and physiotherapists [28]. Remote access to this simulation supported by purposefully designed learning activities may enhance knowledge and skill development for remote x-ray operators in areas relevant to their radiographic scope of practice.

### Study limitations

This study explored the students’ experiences of virtual radiography in the undergraduate pre-clinical radiography course as part of a university chiropractic program.

Due to the small sample size associated with this pilot study, caution should be adopted in interpreting and generalising the results. It is recommended that the study be repeated with an increased sample size to improve the generalisability of the findings. In addition, this study focussed on the lumbar spine, an area of current radiographic scope of practice for chiropractors. It is recommended that future research extend the areas of radiographic practice utilised with this simulation. It is also recommended that future research be extended to include the views of educators.

Previous authors have noted that student performance may be influenced by a variety of factors [8, 23] and this interplay of factors may confound the ability to independently evaluate the role of specific potential enablers such as simulated teaching. This is particularly true when an innovative teaching tool or strategy is introduced into a new learning environment [23].

Future research could examine student usage patterns of innovative teaching programs such as simulated radiography providing valuable information on how to best achieve flexibly-delivered clinical education programs such as this.

## Conclusion

The Projection VR™ software was adapted for use in an undergraduate university chiropractic radiography program.

The introduction of this software was associated with multiple benefits including an increase in skills and confidence in students’ ability to effectively prepare their patients for radiographic positioning and quality imaging while minimising their exposure to irradiation dose.

The software also promoted their problem-solving skills in preparation for their transition to professional practice.

The results of this pilot study are promising and suggest that more extensive testing of the impact of simulated radiography with larger sample sizes and involving a number of chiropractic institutions be considered for further investigation.

## Supplementary Information

**Additional file 1.** (Virtual Radiographic Chiropractic). (Survey to describe Chiropractic students’ experiences on the use of virtual radiography simulation: A pilot observational study).

## Data Availability

The datasets used and/or analysed during the current study are available from the corresponding author on reasonable request.

## Abbreviations

RMIT: Royal Melbourne Institute of Technology
SHBS: School of Health and Biomedical Sciences
