# Supplementary material for: Chiropractic students’ experiences on the use of virtual radiography simulation: a pilot observational study
Source: BMC Med Educ. 2021 Jul 28;21:404. doi: 10.1186/s12909-021-02827-0 (PMC8317307; doi:10.1186/s12909-021-02827-0)
Supplement: Supplementary file 1 — Additional file 1. (Virtual Radiographic Chiropractic). (Survey to describe Chiropractic students’ experiences on the use of virtual radiography simulation: A pilot observational study). [file 12909_2021_2827_MOESM1_ESM.pdf]

Virtual Radiography Chiropractic

Default Question Block

Block Options

Q1

Participant Information Statement to students enrolled in XXXXXXXXXX (XXXX)
Ethics Approval granted by CHEAN Ethics Approval Number ASEHAPP 10-16

Research into Teaching and Learning is a core activity at RMIT University which aims to enhance educational practice. In 2016 we are introducing Virtual Radiography^{TM}, a computer-based simulation into the learning activities in XXXXXXXXXXXXXXXs (XXXXX). To evaluate this introduction of Virtual Radiography^{TM}, we want to collect feedback from you using this anonymous online survey.
You are able to decide if you want to or do not want to participate in this research. Your decision to participate or not participate in this research will not impact on student grades in these courses.
The Participant Information Sheet is available on BlackBoard and also available through the link below. It is important that you read and understand this sheet before participating in the study.

Please read the full Participant Information Sheet

I have read the Participant Information Sheet and I agree to participate in this study.

- Yes, I agree

Q2

In relation to using computer technology, how would you describe yourself?

- Confident
- Moderately confident
- Somewhat confident
- Not at all confident

Q3

Before using Virtual Radiography^{TM}, had you used (hands on experience) computer-based simulation?

- Yes
- No

Display This Question:
If Before using Virtual RadiographyTM, had you used (hands on experience) computer-based simulation? Yes Is Selected

Q4

Before using Virtual Radiography^{TM} how would you describe yourself using computer-based simulation?

- Confident
- Moderately confident
- Somewhat confident
- Not at all confident

Page Break

Q5

These statements are concerned with ease of use and your access to *Virtual Radiography^{TM}*.
Please indicate your level of agreement with each of the following statements

	Strongly agree	Agree	Neither agree or disagree	Disagree	Strongly Disagree
Virtual Radiography is easy to use	○	○	○	○	○
I could control the equipment as I needed when using Virtual Radiography	○	○	○	○	○
I liked using Virtual Radiography	○	○	○	○	○
Technical problems made using Virtual Radiography difficult	○	○	○	○	○
The Virtual Radiography laboratory activity sheet was easy to follow	○	○	○	○	○

Page Break

Q6

The statements are concerned with how *Virtual Radiography*^{TM} may have affected your learning.
Please indicate your level of agreement with each of the following statements
Using Virtual Radiography^{TM} has:

	Strongly agree	Agree	Neither Agree nor Disagree	Disagree	Strongly Disagree
Allowed me to quickly see images and understand if changes needed to be made	○	○	○	○	○
Helped me learn as I was able to repeat activities until I was satisfied with the results	○	○	○	○	○
Encouraged me to think more about radiographic procedures	○	○	○	○	○
Encouraged me to think more about radiographic image evaluation	○	○	○	○	○
Helped me understand the effect of changing radiographic exposure factors on patient dose	○	○	○	○	○
	Strongly agree	Agree	Neither Agree nor Disagree	Disagree	Strongly Disagree
Encouraged me to solve problems	○	○	○	○	○
Had a positive effect on **my ability to evaluate** radiographic images	○	○	○	○	○
Had a positive effect on my **ability to set-up** a radiographic examination	○	○	○	○	○
Had a positive effect on my **confidence level in evaluating** radiographic images	○	○	○	○	○
Had a positive effect on my **confidence level in to set-up** a radiographic examination	○	○	○	○	○

Page Break

Q7

The statements are concerned with your opinion of the use of *Virtual Radiography*^{TM} in laboratory in XXXXXXXX (XXXXXXXX)
Please indicate your level of agreement with each of the following statements

	Strongly agree	Agree	Neither Agree nor Disagree	Disagree	Strongly Disagree
Virtual Radiography complements other learning activities in laboratory	○	○	○	○	○
Virtual Radiography is a valuable part of laboratory	○	○	○	○	○
Virtual Radiography should continue to be used in laboratory	○	○	○	○	○

Q8

Having used Virtual Radiography^{TM} how would you describe yourself using computer-based simulation?

- Very confident
- Moderately confident
- Somewhat confident
- Not at all confident

Page Break

Q9

My age group is

- 18-21 years
- 22-25 years
- 26-29 years
- 30+ years

Q10

My gender is

- Female
- Male

Page Break

Q12

How did using Virtual Radiography^{TM} support your learning inXXXX (XX)?

Q13

Is there anything you would change to improve using Virtual Radiography^{TM} as part of the laboratories in XXXXXXX (XXXXXX)?

Q27

Where else, if at all, do you think Virtual Radiography^{TM} may be useful within the Chiropractic program?

Q14

What did you like most about Virtual Radiography^{TM} simulation?

Q15

What did you like least about Virtual Radiography^{TM} simulation?

Q16

Thank you for providing feedback on the introduction of Virtual Radiography^{TM} into XXXXXXXXXXX. Is there anything else you would like to add?

Add Block

RMIT UNIVERSITY Qualtrics.com Contact Information Legal
